# Post-infectious Inflammatory Motor-Predominant Neuropathy Following Mycoplasma Infection

**DOI:** 10.7759/cureus.74657

**Published:** 2024-11-28

**Authors:** Anush K Shashidhara, Sithu Bala, Stefan Browne, Michael Cliff, Patrick Mallia

**Affiliations:** 1 General Internal Medicine, Royal Free London NHS Foundation Trust, London, GBR; 2 Neurology, Royal Free London NHS Foundation Trust, London, GBR; 3 Respiratory Medicine, Royal Free London NHS Foundation Trust, London, GBR

**Keywords:** immune, infection, motor, pneumonia, polyneuropathy

## Abstract

We present a case of a 37-year-old gentleman diagnosed with post-infectious Guillain-Barré syndrome (GBS) secondary to a Mycoplasma pneumoniae infection. This case highlights the subclinical presentation of neurological symptoms, often overlooked as a complication of M. pneumoniae infection. The patient exhibited significant neurological deficits following initial respiratory symptoms, demonstrating the need for awareness of these potential extrapulmonary complications in clinical practice.

## Introduction

Mycoplasma pneumoniae is a notable pathogen responsible for respiratory infections, particularly atypical pneumonia [1], with epidemics occurring approximately every four to seven years [2]. This bacterium is implicated in approximately 20% of cases of community-acquired pneumonia and has also been implicated in some hospital-based epidemics. Respiratory infection occurs mainly in children and young adults and is often seen in closed community settings, such as schools, universities, nursing homes, and military bases [2].

M. pneumoniae has been associated with severe extrapulmonary manifestations, affecting multiple organs, including neurological disorders such as meningitis, meningoencephalitis, and polyneuropathy [3]. Guillain-Barré syndrome (GBS), is the most common and most severe acute motor neuropathy in the world, affecting around 100,000 people annually [4]. GBS represents a spectrum of disease with a variety of subtypes and disease processes, with the classic subtype being acute inflammatory demyelinating polyneuropathy [5,6].

## Case presentation

A 37-year-old Caucasian male presented to the Emergency Department with a two-week history of productive cough, shortness of breath, and fever and a shorter, one-day history of difficulty passing urine. He was a non-smoker with no significant medical history or prior respiratory conditions. His recent travel history included a visit to an urban seaside town in Spain, with no reported rashes or insect bites and no known unwell contacts.

**Figure 1:**
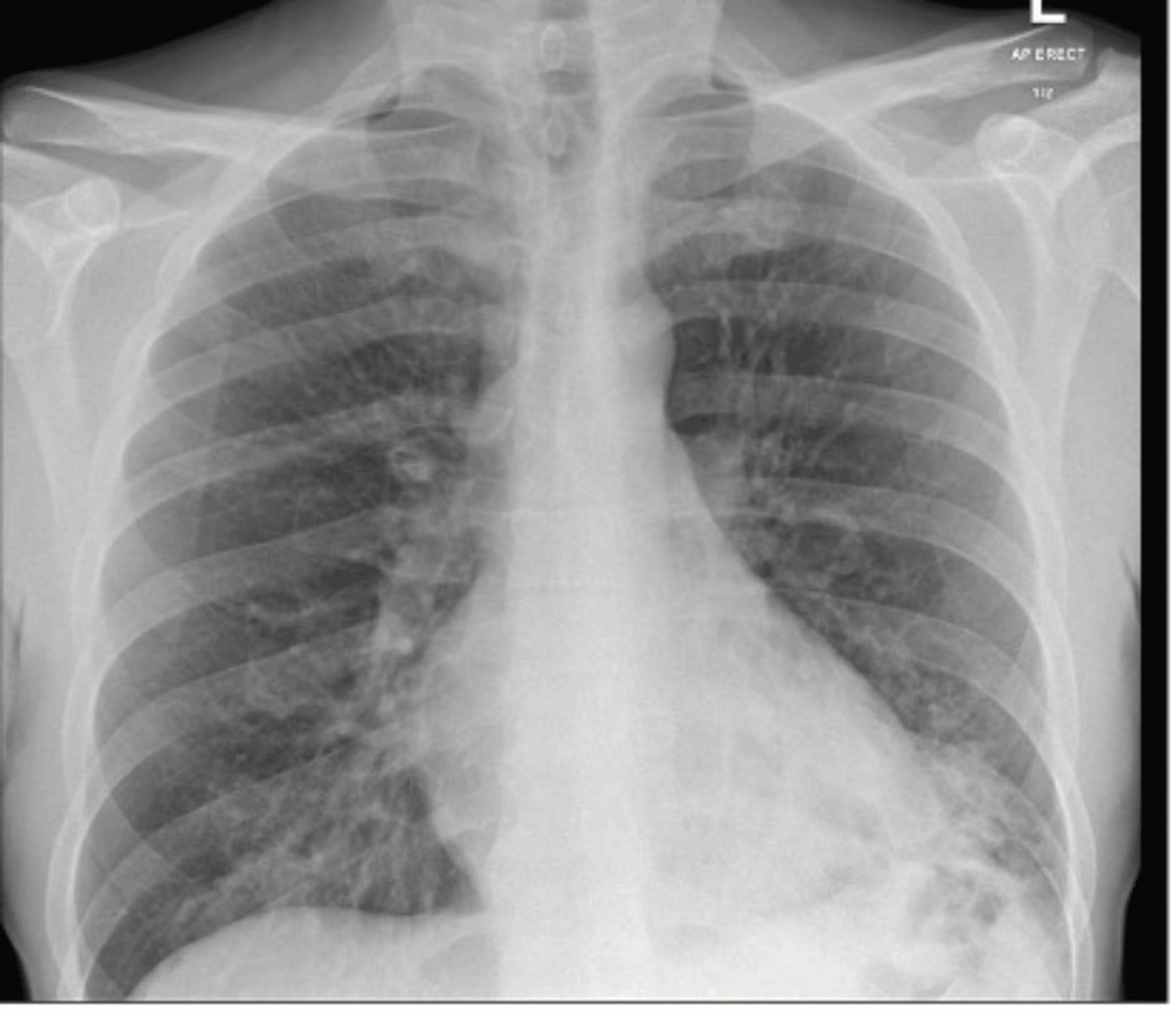
Chest radiograph at initial presentation showing left lower lobe consolidation

**Figure 2:**
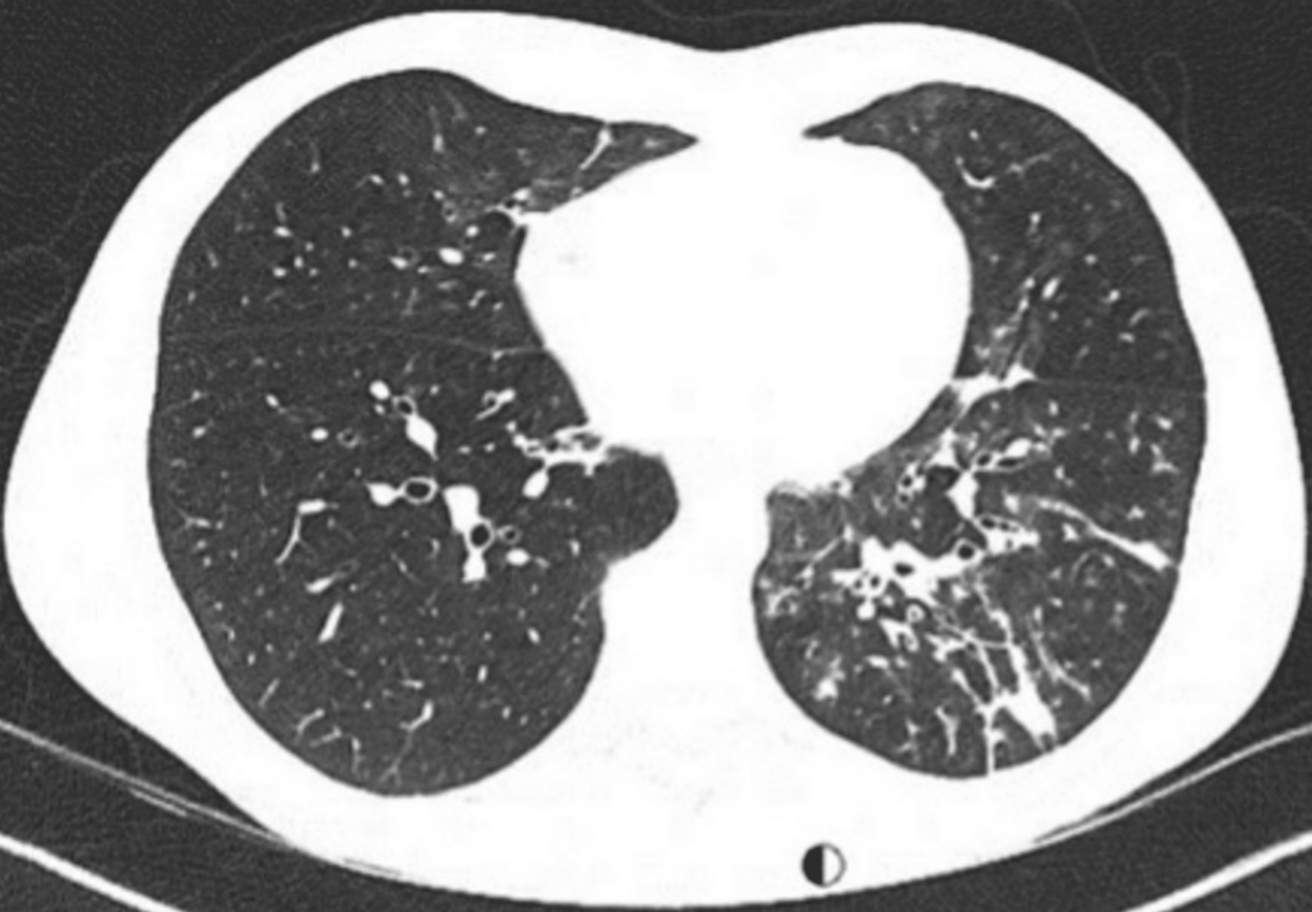
CT pulmonary angiogram showing left lower lobe and to a lesser degree right lower lobe consolidation with airway mucus plugging, diffuse nodularity, and a mild degree of ground-glass change within both lower lobes, worse in the left.

Upon admission, the patient was diagnosed with multifocal pneumonia, confirmed as M. pneumoniae positive via serology (IgG and IgM), with the rest of his blood tests shown in Table 1. A chest x-ray (Figure 1) showed left lower lobe consolidation with some volume loss and tenting of the hemidiaphragm and faint opacification within the right lower lobe. The D-dimer level was elevated to 977ng/mL, prompting the request for a computed tomography pulmonary angiogram (Figure 2), which showed no evidence of pulmonary embolism but revealed multi-focal consolidation with airway mucus plugging. There was also diffuse nodularity, and a mild degree of ground-glass change within both lower lobes, worse within the left.

**Table 1:** Laboratory analysis of blood tests at admission

Test	Value	Normal laboratory range
Hemoglobin	140	135-170g/L
White cell count	12.8	3.5-11x10^9^/L
Platelets	483	140-400 x10^9^/L
Neutrophils	11.0	1.7-7.5x10^9^/L
Lymphocytes	1.0	1-4x10^9^/L
C-reactive protein	54	0-5mg/L
Sodium	132	133-146mmol/L
Potassium	4.3	3.5-5.3mmol/L
Urea	4.3	2.1-7.1mmol/L
Creatinine	74	59-104 microgram/L
Mycoplasma IgG	Positive	
Mycoplasma IgM	Positive	

Upon presentation, a post-void bladder scan revealed a bladder volume of 440 milliliters. Subsequently, a catheter was inserted, with an initial unsuccessful removal 48 hours later, followed by a successful removal at 72 hours. A CT scan of his kidney, ureters, and bladder was performed to identify any structural causes of this gentleman’s urinary retention but only showed a moderately enlarged prostate without obstructive lesions.

During his 72-hour hospital stay, he was treated with oral co-amoxiclav and clarithromycin and was discharged after symptom improvement. He was due to complete a full course of oral antibiotics with a repeat chest radiograph in primary care at six weeks post-discharge.

However, he re-presented to the emergency department seven days after initial discharge with worsening pain, restlessness, and weakness in his legs. The pain was described as aching and was concentrated in the thighs, with occasional transient pins and needles in his feet. Additionally, he reported unusual sensations on the left side of his face, including eye-watering and altered facial movement.

Neurological examination revealed bilateral lower motor neuron facial weakness with bilateral orbicularis oculi weakness, normal upper limb strength, and tone. Lower limb strength was decreased globally, proximally more so than distally, with reduced tone. Biceps, triceps, and brachioradialis reflexes were normal, with a notable reduction in the right knee reflex and absent ankle jerks. Pinprick, light touch, vibration sensation, and joint position sense were normal throughout the upper and lower limbs.

Cerebrospinal fluid (CSF) analysis (table 2) showed elevated CSF protein (3.68 g/L), and a raised white cell count of 26/mm³ (100% lymphocytes), with no bacterial growth. A comprehensive viral panel (CSF viral PCR) was negative for herpes simplex virus (HSV), varicella-zoster virus (VZV), enterovirus, and parechovirus. Immune-related peripheral neuropathy screen was negative (table 3).

**Table 2:** Cerebrospinal fluid analysis HSV - herpes simplex virus, VZV - varicella-zoster virus

Test	Result
Appearance	Clear, colorless fluid
Opening pressure	18 cmH2O (10-20)
White blood cell count	26 per mm3
Polymorphs	0.0% No polymorphs
Lymphocytes	100.0%
Red blood cell count	<1,000mm3
Gram stain	No organisms seen
Culture	No growth
Protein	3.68 g/L (0.15-0.45)
Lactate	2.1 mmol/L (1.1-2.4)
Oligoclonal bands	Negative (both serum and CSF)
Parechovirus RNA	Not detected
HSV DNA	Not detected
VZV DNA	Not detected
Enterovirus RNA	Not detected

**Table 3:** Peripheral neuropathy antibody screen

Test	Result
IgG GM1 Ab level	Negative
IgM GM1 Ab level	Negative
IgG GM2 Ab level	Negative
IgM GM2 Ab level	Negative
IgG GD1a Ab level	Negative
IgM GD1a Ab level	Negative
IgG GD1b Ab level	Negative
IgM GD1b Ab level	Negative
IgG GQ1b Ab level	Negative
IgM GQ1b Ab level	Negative

**Figure 3:**
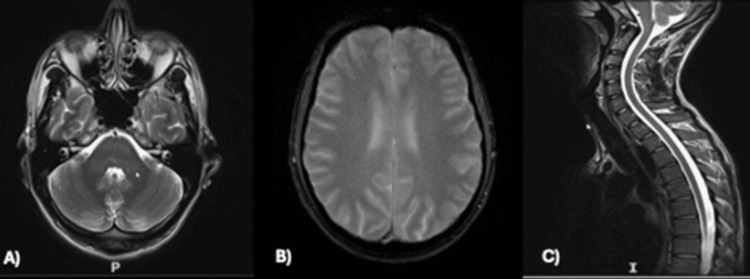
Normal magnetic resonance imaging of the patient's brain and spine, with no evidence of demyelination, hemorrhage, or infarct. (A) MRI head T2-weighted sequence, (B) MRI head susceptibility-weighted imaging (SWI) sequence, and (C) MRI spine.

Non-contrast MRI of the brain and the whole spine (Figures 3A-3C) ruled out any underlying structural pathology.

Electromyography (EMG) undertaken 10 days following the onset of his neurological symptoms confirmed a pure-motor inflammatory demyelinating neuropathy (Tables 4-7), characterized by delayed/absent blink reflexes and marked conduction slowing, with prolonged F-latency suggesting demyelination targets near the radicular level.

**Table 4:** Electromyography results

Results	
Right orbicularis oculi	No spontaneous activity. Individual units of normal configuration. Units firing at high rates in relative isolation in a severely reduced interference pattern to 2mV.
Right orbicularis oris	No spontaneous activity. Individual units of normal configuration. Units firing at high rates in relative isolation in a moderately reduced interference pattern to 2mV.
Right rectus femoris	No spontaneous activity. Individual units of normal configuration. Units firing at high rates in relative isolation in a moderately reduced interference pattern to 3mV.
Right tibialis anterior	No spontaneous activity. Individual units of normal configuration. Units firing at high rates in relative isolation in a moderately reduced interference pattern to 3mV.
Right gastrocnemius (lateral head)	No spontaneous activity. Individual units of normal configuration. Units firing at high rates in relative isolation in a mildly reduced interference pattern to 3mV.

**Table 5:** Facial/blink reflex study

Reflex/laterality	Right	Left
Ipsilateral R1	17 ms	18 ms
Ipsilateral R2	47 ms	0 ms
Contralateral R2	0 ms	0 ms

**Table 6:** Sensory action potentials

Sensory action potentials	Amplitude	Conduction velocity
	(µV)	(m/s)
Right median (F2-wrist)	10	52
Right median (F3-wrist)	16	51
Right ulnar (F5-wrist)	5.6	47
Right sural (calf-ankle)	12	51
Right sup peroneal (calf-ankle)	3.8	42

**Table 7:** Motor conduction velocities

Median (stimulation electrode on abductor pollciis brevis)	Right
Distal motor latency	4.2 ms
Conduction velocity (wrist-elbow)	43 m/s
Motor amplitude potential (wrist)	9.2 mV
Motor amplitude potential (elbow)	7.6 mV
F latency	37 ms
Ulnar (stimulation electrode on ADM)	Right
Distal motor latency	3.1 ms
Conduction velocity (wrist-below elbow)	48 m/s
Conduction Velocity (around elbow)	48 m/s
Motor amplitude potential (wrist)	10 mV
Motor amplitude potential (below elbow)	8.4 mV
Motor amplitude potential (above elbow)	8.4 mV
F latency	39 ms
Common peroneal (stimulation electrode on EDB)	Right
Distal motor latency	6.0 ms
Conduction velocity (fib neck-ankle)	41 m/s
Motor amplitude potential (ankle)	1.6 mV
Motor amplitude potential (fib neck)	1.6 mV
Posterior tibial (stimulation electrode on AH)	Right
Distal motor latency	7.1 ms
Motor amplitude potential (ankle)	5.1 mV
F latency	76 ms

Overall, his presentation, clinical examination findings, and investigations were felt to be consistent with a motor variant of GBS secondary to Mycoplasma infection. A mildly raised CSF white cell count in GBS, though uncommon, has been documented in several studies.

The patient was closely monitored in an enhanced medical care unit and received a five-day course of intravenous immunoglobulin (IVIG). IVIG was commenced on day 10 since the onset of his neurological symptoms. His forced vital capacity (FVC) improved significantly from 2.08L to 4.93L indicating recovery in respiratory function. His FVC remained above the threshold for intubation and mechanical ventilation at all points during admission (1.4L, based on a threshold of 20mL/kg and a body weight of 69.1kg on admission). There was also no evidence of type two respiratory failure during his admission, as evidenced by blood gas testing. On discharge, the patient had mild global weakness in his lower limbs with Medical Research Council (MRC) grade 4/5, and full power in the upper limbs but achieved full motor recovery over six months with physiotherapy. 

## Discussion

M. pneumoniae is a frequent cause of atypical pneumonia, with a spectrum of extra-pulmonary manifestations, with neurological sequelae being uncommon, particularly in adults [2,3,7]. GBS classically presents with ascending weakness with a monophasic course - symmetry remains a key diagnostic feature [8,9]. The case we describe is typical in many ways; it presented shortly after the acute mycoplasma infection, with a delay of just days, and progressively worsened with no acute remission of symptoms, while producing an eventual bilateral, flaccid paralysis. 

However, several atypical clinical features were present that initially obscured our diagnosis and delayed the initiation of IVIG therapy. Notably, the erratic muscle involvement, particularly affecting the orbicularis oculi, initial asymmetry of patellar reflexes, and the atypical presenting complaint of restless legs were significant contributing factors to our diagnostic dilemma.

The epidemiological studies indicate the incidence of M. pneumoniae-associated GBS and it was found that M. pneumoniae was detected in about 2%-5% of GBS cases [10]. The mildly raised CSF pleocytosis was unusual but can occur in GBS and was less than 50 cells [11].

EMG and nerve conduction studies play a key role in confirming GBS - by identifying characteristic abnormalities like conduction block in demyelinating forms or reduced motor amplitudes in axonal variants [12]. However, its diagnostic utility is time-sensitive, with EMG findings sometimes being normal within the first week of symptoms due to delayed onset of changes. Repeat testing is often necessary in cases of high clinical suspicion.

Our findings align with the diagnostic criteria for GBS as outlined by The European Academy of Neurology (EAN) and the Peripheral Nerve Society in their 2023 guidelines, as well as the Brighton Criteria developed in 2011, which helps stratify GBS diagnosis through clinical and testing criteria [8,9,13]. The CSF analysis showing disproportionately elevated protein relative to the white cell count, and EMG findings of motor nerve demyelination, supported the diagnosis, in addition to the clinical findings present.

**Table 8:** Brighton Criteria for Diagnosis of GBS +, present; -, absent; +/-, present or absent; GBS, Guillain-Barré syndrome; NCS, nerve conduction studies. *If CSF is not collected or results not available, nerve electrophysiology results must be consistent with the diagnosis of Guillain- Barré syndrome. Level 1 is the highest level of diagnostic certainty; Level 4 is the lowest level of diagnostic certainty.

Diagnostic criteria	1	2	3	4
Bilateral and flaccid weakness of limbs	+	+	+	+/-
Decreased or absent deep tendon reflexes in weak limbs	+	+	+	+/-
Monophasic course and time between onset-nadir 12 h to 28 days	+	+	+	+/-
CSF cell count < 50/ μl	+	+*	-	+/-
CSF protein concentration > normal value	+	+/-*	-	+/-
NSC findings consistent with one of the subtypes of GBS	+	+/-	-	+/-
Absence of alternative diagnosis for weakness	+	+	+	+

An interesting point of note in our case is the initial presentation with urinary retention - autonomic dysfunction is considered a supporting feature of GBS diagnosis within the 2023 guidelines, and a case series of 65 patients in Japan in 2009 showed that almost a third of their patients had urinary dysfunction, and almost 10% exhibited urinary retention [9,14]. 

Spirometry remains an essential tool for the monitoring of GBS, especially with the risk of respiratory muscle involvement, with systematic reviews showing a relationship between spirometry and the need for ventilation, although there is limited evidence for specific thresholds [15]. In the United Kingdom, we often use an FVC threshold of below 20mL/kg of body weight to guide the need for intubation with mechanical ventilation. Serial single breath counting has also been found to be a useful and cost-effective monitoring tool when used as a surrogate marker [16]. Respiratory failure requires the need for specialist intensive care or high dependency input for consideration of mechanical ventilation modalities [9,17]. 

The prognosis of GBS varies greatly, with around one-third of patients having incomplete recovery, with high age of onset and diarrhea preceding onset both being associated with a lower rate of independent walking at various time frames up to six months [9,18]. IVIG and plasma exchange are both treatments available that have been shown to reduce disability after a month of symptom onset [9,19]. The prognosis of mycoplasma-induced GBS compared to other causes of GBS is not well studied.

## Conclusions

This case illustrates the potential for neurological complications arising from M. pneumoniae infections, specifically the occurrence of GBS. The atypical presentation, including leg pain without weakness, urinary retention, and facial involvement, underscores the necessity for awareness among clinicians regarding the spectrum of complications following Mycoplasma infections. Timely recognition, investigations, and appropriate treatment, such as IVIG, can lead to favorable outcomes. Further investigation is needed into the prognostication of mycoplasma-associated GBS compared to other causes of the syndrome.
